# Technological Optimization and Antioxidant Efficacy via the NRF-2-Mediated Defense Pathway of *Corylus avellana* L. Skin Extracts: A Sustainable Approach for Developing Health-Promoting Natural Products

**DOI:** 10.3390/ph19040539

**Published:** 2026-03-27

**Authors:** Immacolata Faraone, Maria Ponticelli, Claudia Mangieri, Ilaria Nigro, Ludovica Lela, Antonio Vassallo, Carlo Cosentino, Nikolay T. Tzvetkov, Vittorio Carlucci, Maria Francesca Armentano, Luigi Milella

**Affiliations:** 1 Department of Health Sciences, University of Basilicata, Via dell’Ateneo Lucano 10, 85100 Potenza, Italy; immacolata.faraone@unibas.it (I.F.); maria.ponticelli@unibas.it (M.P.); ilaria.nigro@unibas.it (I.N.); vittorio.carlucci@unibas.it (V.C.); mariafrancesca.armentano@unibas.it (M.F.A.); luigi.milella@unibas.it (L.M.); 2 Department of Basic and Applied Sciences, University of Basilicata, Via dell’Ateneo Lucano 10, 85100 Potenza, Italy; claudia.mangieri@unibas.it; 3 Department of Agriculture, Forest, Food and Environmental Sciences, University of Basilicata, Via dell’Ateneo Lucano 10, 85100 Potenza, Italy; carlo.cosentino@unibas.it; 4 Department of Biochemical Pharmacology & Drug Design, Institute of Molecular Biology “Roumen Tsanev”, Bulgarian Academy of Sciences, Acad. G. Bonchev Str., Bl. 21, 1113 Sofia, Bulgaria; ntzvetkov@gmx.de; 5 Institute of Genetics and Animal Biotechnology, Polish Academy of Sciences, 05-552 Magdalenka, Poland

**Keywords:** *Corylus avellana* L. skins, polyphenols, antioxidant activity, NRF-2 signaling pathway, intracellular ROS, ultrasound-assisted extraction, full factorial design, agro-industrial by-products, nutraceutical ingredients

## Abstract

**Background/Objectives**: The valorization of bioactive compounds from food industry by-products aligns with sustainable development goals and represents a strategy for obtaining functional ingredients. Hazelnut (*Corylus avellana* L.) skins are a phenolic-rich residue with high antioxidant potential, but their extraction conditions and cellular mechanisms of action remain insufficiently explored. **Methods**: Ultrasound-assisted extraction was optimized using a 3^3^ Full Factorial Design (FFD) by investigating temperature (30–50–70 °C), extraction time (1–2–3 h), and solvent composition (water/ethanol). Antioxidant activity was evaluated using multiple *in vitro* assays, including Total Phenolic Content (TPC), DPPH, ABTS, FRAP, and *β*-carotene bleaching (BCB) assays. The optimized extract (OE) was chemically characterized by UHPLC–MS/MS and its activity was evaluated in HepG2 cells for biocompatibility, modulation of intracellular ROS levels, and antioxidant pathway activation. **Results**: Optimal extraction conditions were identified as 30 °C, 70.86 min (1.181 h), and 21.13% ethanol (*v*/*v*), yielding an extract with enhanced antioxidant capacity. UHPLC–MS/MS analysis revealed 25 bioactive compounds, mainly flavonoids and phenolic acids, relevant for oxidative stress modulation. The extract significantly reduced tert-butyl hydroperoxide (TBH)-induced intracellular ROS levels, restoring antioxidant proteins involved in the Nuclear Factor erythroid 2-related factor 2 (NRF-2)-mediated defense pathway. **Conclusions**: The optimized hazelnut skin extract combines strong antioxidant efficacy with cellular compatibility, supporting its potential application as a functional ingredient for nutraceutical and pharmaceutical strategies targeting oxidative stress-related conditions.

## 1. Introduction

**Figure 1:**
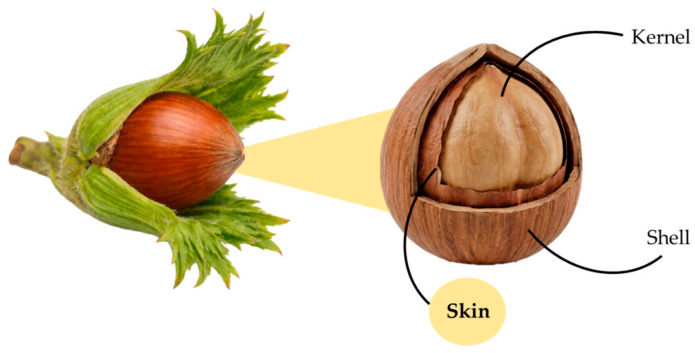
The morphological structure of the hazelnut.

In the contemporary era, the global annual production of vegetable waste from industrial processes has exceeded 200 million tons [1]. A significant proportion of this waste is used as animal feed, fertilizer, and fuel, or released directly into the environment without further use [2]. However, research findings demonstrate that these substances can still serve as a source of fibers, specialized metabolites, lipids, and proteins, representing an interesting source of bioactive compounds. In fact, their utilization has become the fundamental principle of the circular economy [3], which is a sustainable development model that aims to minimize waste and environmental impact by maximizing the use of technological resources and reintegrating biological resources. It is founded on the principles of reuse and recycling by minimizing the production of new resources and the disposal of secondary raw materials [3,4]. This approach stands in contrast to the conventional linear economic model, which is oriented toward the acquisition of raw materials, production, transformation into finished products, and disposal, without any support for sustainable development [5]. Therefore, to reduce pressure on the environment and improve the security of the supply of primary raw materials, a shift from a linear economy to a circular one is mandatory [6]. In particular, the increasing demand for sustainable and circular approaches in agri-food systems has stimulated growing interest in the valorization of agricultural by-products [7]. Among these, hazelnut processing generates large amounts of solid residues, including shells, skins, leaves, and husks [8]. The scientific name of the European hazelnut is *Corylus avellana* L., a plant species belonging to the Betulaceae family. It is cultivated in several countries, with Turkey being the leading producer, followed by Italy, China, and others. Hazelnut, generally eaten raw or roasted, is commonly used in cakes, chocolates, and other preparations and snacks [9]. Simultaneously, it serves as an important source of nutrients, including proteins, vitamins, amino acids, fatty acids, and specialized metabolites [10]. In this context, the present work aims to investigate the chemical composition of hazelnut skin, with particular attention paid to its phenolic content and associated bioactivities. Hazelnut skin (Figure 1) is the brown covering removed during the roasting process due to its bitter taste, accounting for approximately 2.5% of the total hazelnut kernel weight [11,12]. The skin contains 14.5% fat, 8% protein, and 7.5% moisture. Dietary fiber (67.7%) represents the main content, of which the majority is insoluble fiber (57.7%). Moreover, the skin contains about 27% of polyphenols that influence antioxidant activity [13]. Every year, the agri-food industry produces large quantities of hazelnut skin as a by-product of processing. Different scientific studies have indicated that the phenolic compounds of the nut are principally concentrated in the skin [11].

Numerous studies have explored the antioxidant properties of hazelnut skin, often considered a by-product of the hazelnut industry, with a focus on enhancing its economic value [12,14]. For this reason, it is important to optimize the extraction process. The objective and principal novelty of this study was to develop an optimized hazelnut skin extract using a 3^3^ full factorial experimental design (FFD). The FFD was developed to maximize the extraction of specialized metabolites with antioxidant activity. This approach enabled the identification of optimal extraction conditions to maximize antioxidant potential using green solvents and employing hazelnut skin from plants grown in the Basilicata region and in the Viterbo area, highlighting the influence of the production site on extracts’ characteristics. In addition, the optimized extract was investigated for its phytochemical profile and antioxidant activity, evaluated by using spectrophotometric assays and HepG2 cells as a cellular model. Specifically, *in vitro* assays were performed to assess cytotoxicity, the reduction in intracellular ROS under pro-oxidant stimulation, and the modulation of key proteins involved in the Nuclear Factor erythroid 2-related factor 2 (NRF-2)-mediated antioxidant response, such as SOD2, CAT, and NQO1. In conclusion, this experimental design aimed to link extraction optimization, phytochemical profiling, and mechanism-oriented cellular assays, thereby supporting the development of a waste by-product with promising nutraceutical and pharmaceutical applications targeting oxidative stress-related disorders [15].

## 2. Results

### 2.1. Model Adequacy

This study focuses on obtaining optimized hazelnut skin extracts with the highest phenolic content and antioxidant activity. Within this scope, the effects of solvent (100–50–0% EtOH), temperature (30–50–70 °C), and time (1–2–3 h) on hazelnut skin extracts’ total phenolic content and antioxidant activity (as assessed by DPPH, ABTS, FRAP and *β*-Carotene Bleaching (BCB) assays) were investigated using a 3^3^ full factorial experimental design implemented with three replications of the central point.

**Figure 2:**
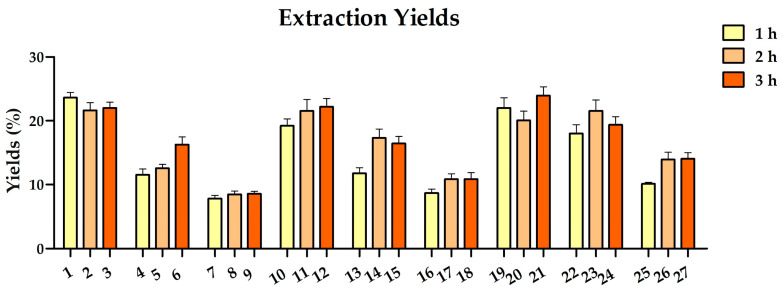
Extraction yields for the 27 extracts of hazelnut skin obtained.

The extraction yield is a key parameter for evaluating the efficacy of the extraction process employed. As demonstrated in the results reported in Figure 2, the extraction yield was influenced by the experimental conditions or independent variables (Table 1). Specifically, the highest extraction yield was obtained in samples extracted at different temperatures and times in 100% ethanol, reaching values above 20%, while yields between 8 and 14% were observed for extractions in 100% H_2_O. Conversely, samples extracted in 50% ethanol obtained yields ranging from 11 to 20%, exhibiting a proportional trend with the increase in temperature.

The complete experimental design is described in detail in Table 1, while the design matrix and ANOVA results are reported in Tables S1 and S2, respectively.

Correlation analysis among the different antioxidant assays (TPC, DPPH, ABTS, FRAP, and BCB) showed consistent trends, supporting the reliability of the antioxidant evaluation, although variations were observed due to the different reaction mechanisms involved.

The statistical analysis was conducted on the raw data without applying any transformations, such as logarithmic or square-root transformations. As shown in Table S1, the statistically significant *p*-values indicate that the selected independent variables significantly influence the response variables. These results are further supported by the fit statistics presented in Table S2, which show regression coefficients (R^2^) of 0.97, 0.98, 0.98, 0.97, and 0.91 for TPC, ABTS, DPPH, FRAP, and BCB, respectively. Even more significant is the high congruence between the adjusted R^2^ (Adj-R^2^) and the predicted R^2^ (Pred-R^2^). For all variables, the difference between the two values is less than 0.2, confirming that the model is not overfitting and has excellent predictive ability within the experimental domain. Furthermore, the coefficient of variation (CV%) was low, 7.13%, 6.72%, 6.32%, 7.33%, and 20.48% for TPC, ABTS, DPPH, FRAP, and BCB, respectively (Table S3), indicating high analytical precision. Finally, the Adequate Precision obtained, representing the signal-to-noise ratio measurements, is also noteworthy. A value greater than 4 is considered the minimum required to use the model in navigating the experimental space [16,17,18]. In this case, the achieved Adequate Precision ranged from 14.95 to 24.26, indicating a very strong signal relative to background noise. To ensure the statistical integrity of the model, the distribution of residuals was also examined using Normal Plots of residuals. As shown in Figure S1, the residuals are distributed around the center line, confirming that the model fits the experimental data adequately and follows a normal distribution. Following this validation, optimization was then performed using the results obtained from TPC, DPPH, FRAP, ABTS, and BCB as dependent variables.

### 2.2. A Study of the Effect of Independent Variables on Dependent Variables

To quantify how each independent variable influenced the dependent variables, a second-degree polynomial equation was applied to all factors. These mathematical models allowed for the prediction of specific responses based on the chosen factor levels, effectively mapping the impact of each variable, as detailed in Table 2.

Specifically, analysis of variance (ANOVA) applied to quadratic models identified the influence of the independent variables [ethanol concentration (A), temperature (B), and extraction time (C)] on phenolic content and antioxidant properties of hazelnut skin extracts. Among the dependent variables analyzed (TPC, ABTS, DPPH, FRAP, and BCB), ethanol concentration was the most critical factor, showing an extremely significant negative linear effect (*p*-value < 0.0001). The high linear coefficients (e.g., −1273.86 for DPPH, −766.722 for FRAP, and −552.82 for ABTS) indicate that an excessive increase in ethanol concentration significantly reduces extraction efficiency, likely due to the reduced polarity of the solvent system compared to hydroalcoholic mixtures. In addition to the linear effect, a highly significant quadratic term (A^2^) is observed for all parameters. The negative values of these coefficients confirm the downward curvature of the response surface, suggesting an optimum point in an intermediate range of hydroalcoholic mixtures, where the synergy between water and ethanol favors the solubilization of a broader spectrum of phytochemicals.

**Figure 3:**
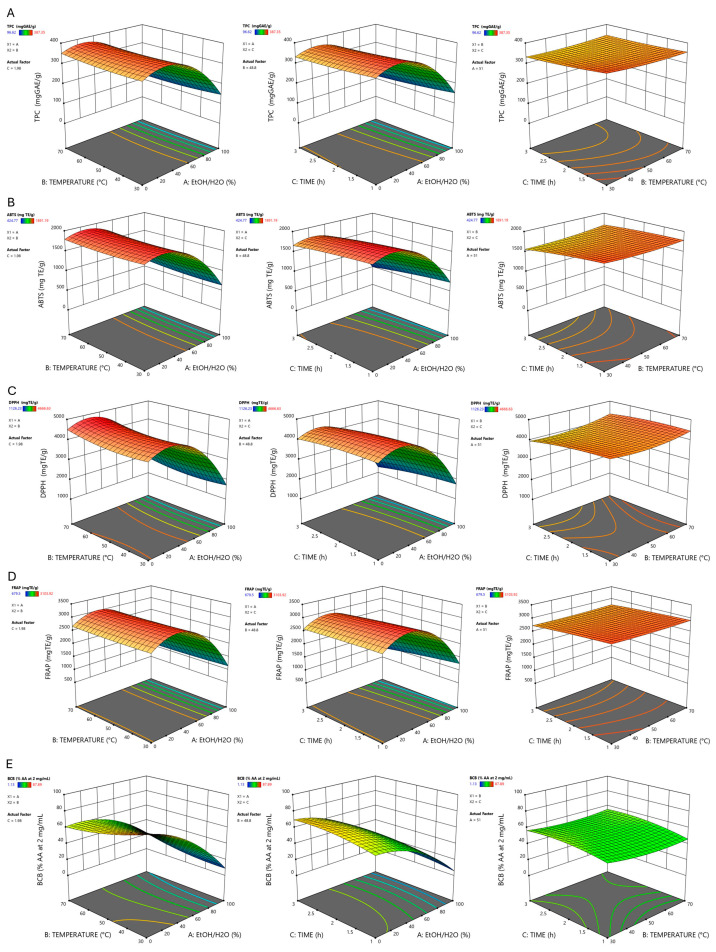
Surface plots with the relative contour plots for Total Phenolic Content (TPC) (**A**), 2,2-diphenyl-1-picrylhydrazyl assay (DPPH) (**B**), 2,2′-azino-bis(3-ethylbenzothiazoline-6-sulfonic acid) assay (ABTS) (**C**), Ferric Reducing Antioxidant Power assay (FRAP) (**D**), and *β*-Carotene Bleaching assay (BCB) (**E**).

Extraction time (C) had a significant impact on phenolic yield (TPC; *p*-value = 0.0112) and on antioxidant capacities measured by ABTS, DPPH, and FRAP (*p*-value = 0.0007, 0.0221, and 0.0146, respectively). The negative sign of the coefficient for C suggests that prolonged contact times may induce thermal or oxidative degradation of the extracted compounds. In contrast, temperature (B) did not show significant linear effects in most tests, except for DPPH (*p*-value = 0.0378). In this case, the positive linear effect indicates that an increase in thermal energy favors the extraction kinetics of specific molecules that act as radical scavengers. Finally, except for BCB, the interactions among variables were not significant, indicating that the factors act predominantly independently of one another. Figure 3 reports the surface plots with the relative contour plots, which are representative of the influence of each selected variable on the independent ones.

### 2.3. Multiple Response Optimization

The final objective of the optimization process is to determine the specific combination of extraction parameters that yields an extract with the highest phenolic content and antioxidant activity. The model validation identified a hydroalcoholic mixture containing 21.13% ethanol, a temperature of 30 °C, and an extraction time of 70.86 min (1.181 h) as the optimal conditions (desirability = 0.96). These conditions reflect the importance of an intermediate-polarity hydroalcoholic environment and short extraction times to preserve the integrity of the polyphenols, minimizing the thermal degradation phenomena highlighted by the negative coefficients of time (C) and ethanol (A).

Considering the obtained extraction conditions, an optimized extract (OE) was made, obtaining data that fall into the predicted results generated by the model (Table 3).

### 2.4. Evaluation of the Phytochemical Profile

**Figure 4:**
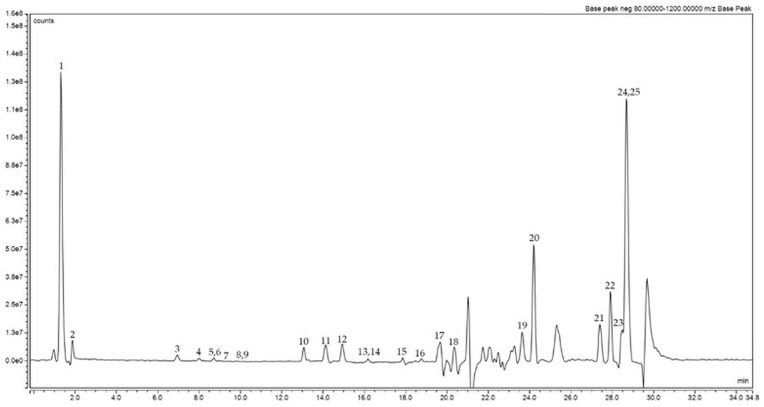
Base peak chromatogram in negative ionization mode (*m*/*z* = 80–1200) for hazelnut skin optimized extract.

A careful analysis of the phytochemical profile of the optimized hazelnut skin extract was performed using UHPLC-MS. The obtained chromatogram is reported in Figure 4.

Twenty-five compounds were detected in negative ionization mode, of which nine were identified and quantified by comparing the retention time, mass spectrum, and fragmentation pattern of the reference standards. Further details are shown in Table 4.

The identified compounds were classified into organic acids (malic acid (**2**)), phenolic acids (3,5-dihydroxybenzoic acid (**3**)), flavonoids (catechin (**6**), procyanidin B2 (**7**), epicatechin (**9**), naringenin (**14**), luteolin (**15**)), dihydrochalcones (phloridzin dihydrate (**11**)), and triterpene (ursolic acid (**22**)). The most abundant compound is 3,5-dihydroxybenzoic acid (**3**), a phenolic acid known for its antioxidant properties, which may contribute to the overall redox modulation activity of hazelnut skin extract, working in synergy with organic acids, such as malic acid (**2**), and flavonoids, such as catechin (**6**), also present in moderate quantities. The findings are consistent with other research on hazelnut skin [19,20], which characterizes this by-product as an important source of flavonoids, thereby validating the efficacy of optimized ultrasound-assisted extraction conditions in enhancing the recovery of these compounds. The prevalence of flavonoids can be attributed to the solvent composition and the moderate extraction temperature; in fact, these factors favor the extraction of moderately polar phenolic compounds, such as flavonoids, while limiting thermal degradation [21]. The flavonoids detected, including procyanidin B2 (**7**), naringenin (**14**), and luteolin (**15**), are well-known for their antioxidant properties and their ability to modulate cellular redox balance [22,23]. It has been reported that these compounds contribute to antioxidant activity through different mechanisms, including activation of intracellular antioxidant pathways [24].

Compounds lacking authentic reference standards were tentatively identified based on accurate mass, isotopic distribution, and MS/MS fragmentation patterns, supported by comparison with literature data. Although these identifications provide a reasonable level of confidence, they should be considered putative.

Among the compounds detected but not identified by comparison with standards, compound **1** was assigned to hydroxycinnamoyl glycosides, namely caffeoyl hexoside, at [M-H]^−^ *m*/*z* 341, resulting in characteristic product ions derived from hexose mass fragmentation in the MS^2^ experiment as described above (base peak fragment ions [M-H-162]^−^). The elimination of water generated additional fragment ions, facilitating the identification of the hydroxycinnamic acid moiety [M-H-162-18]^−^ and CO_2_ [M-H-162-44]^−^. The latter fragmentations yielded product ions at *m*/*z* 179 and 161 [25].

Compounds **4**, **5**, and **8** were characterized by the same [M-H]^−^ ion and a highly similar MS/MS fragmentation pattern of procyanidin B2, so they were tentatively assigned as procyanidin-*type* compounds. As reported by Gu et al. [26], *m*/*z* 577 is indicative of B-*type* procyanidin dimers, and the fragment ions at *m*/*z* 254, 203, and 125 suggest the presence of structural isomers.

Compound **10**, characterized by a precursor ion at *m*/*z* 343, could be tentatively associated with the class of diarylheptanoids. This hypothesis is supported by the similarity in molecular mass and predicted formula to those of known diarylheptanoids, such as carpinontriol B [27] and asadanin, which have been reported to contribute to the bitter off-taste in hazelnuts [28].

Compound **12** was tentatively assigned to azelaic acid, based on its identical precursor ion [M-H]^−^ at *m*/*z* 187 with the predicted molecular formula C_9_H_16_O_4_. The corresponding MS/MS fragmentation pattern shows the characteristic fragment at *m*/*z* 125, as reported [29].

As reported by Babacan et al. [30], compounds **19** and **20** could be tentatively assigned as isomers of dihydroxyoctadecanoic acid, as they share the same [M–H]^−^ precursor ion at *m*/*z* 315, the same predicted molecular formula C_18_H_36_O_4_, and common fragment ions at *m*/*z* 297 and 201. In particular, this latter fragment corresponds to the loss of one molecule of water [M-H-H_2_O]^−^, while the fragment at *m*/*z* 279 originates from the loss of two molecules of water [M-H-2H_2_O]^−^.

Compounds **23** and **24** were tentatively identified as linoleic acid and palmitic acid, respectively. As previously reported [29], a precursor ion at *m*/*z* 279 with a fragment ion at *m*/*z* 261, resulting from the loss of one water molecule, is indicative of linoleic acid. This assignment is further supported by data obtained from PubChem (CID 5280450).

Similarly, compound **24**, showing a precursor ion at *m*/*z* 255 and fragment ions at *m*/*z* 203 and 201, was tentatively assigned as palmitic acid, in agreement with the fragmentation pattern reported in the literature [29] and supported by PubChem data (CID 985). Compound **25**, based on its mass spectral characteristics, was therefore tentatively assigned to the same fatty acid family. Notably, these types of fatty acids have already been reported in the literature as compounds of hazelnut matrices, supporting the possibility of their presence in the optimized hazelnut skin extract [31].

However, further structural confirmation of these compounds through comparison with standards would be required for definitive identification.

The rich flavonoid profile of the optimized extract provides a plausible chemical basis for the high antioxidant capacity observed in the *in vitro* assays. 

### 2.5. Effect of OE on Cell Viability

**Figure 5:**
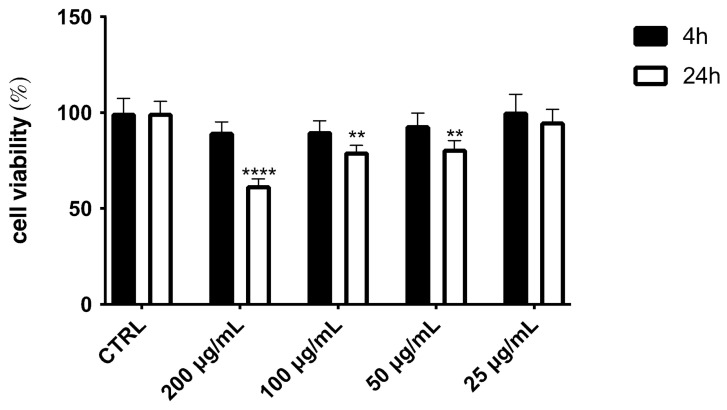
Cell viability, evaluated via MTT assay, of HepG2 cells treated with different concentrations (200–25 μg/mL) of optimized hazelnut skin extract for 4 h and 24 h. Data are expressed as mean ± SD of three independent experiments (*n* = 3) and were analyzed by one-way ANOVA followed by Tukey’s post hoc test. **** *p* < 0.0001 and ** *p* < 0.01 *vs*. control cells (CTRL).

Exposure of HepG2 cells to different concentrations (200–25 μg/mL) of OE induced a dose-dependent reduction after 24 h of exposure (Figure 5), especially at the highest concentration. However, no toxicity was observed after 4 h of treatment. Our results agree with previous data, even though a direct comparison is not possible because different times, concentrations, or cell lines were used [31,32]. The presence of phenols, neolignans, or tannins could explain the observed effect [32].

### 2.6. OE Reduced ROS Levels by Increasing Antioxidant Proteins

The role of *C. avellana* skin as an inhibitor of reactive oxygen species (ROS) was investigated. ROS are highly reactive molecules that contribute to a range of physiological and pathological conditions, including aging, cancer, cardiovascular diseases, and neurodegenerative disorders. Excessive ROS production can lead to oxidative stress, a key factor in the development of various chronic diseases [33]. As such, natural plant-derived compounds that can reduce ROS levels offer promising therapeutic potential.

**Figure 6:**
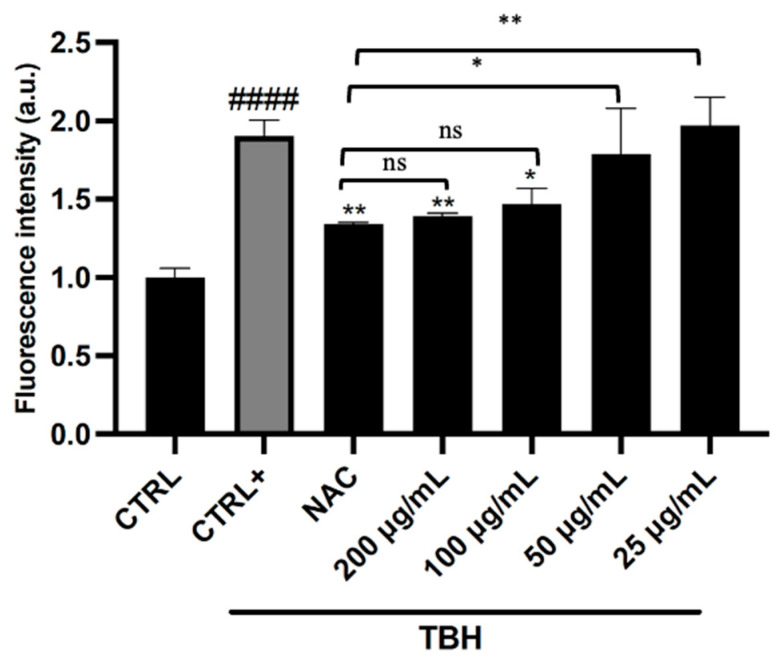
Effect of optimized hazelnut skin extract on intracellular ROS generation in HepG2 cells under TBH stress. Data are expressed as mean ± SD of three independent experiments (*n* = 3) and were analyzed by one-way ANOVA followed by Tukey’s post hoc test. ^####^ *p* < 0.0001 *vs*. CTRL, ** *p* < 0.01, and * *p* < 0.05 *vs*. TBH -treated cells (CTRL+); ns: not significant.

To assess the antioxidant effects of hazelnut skin more thoroughly and to confirm data from *in vitro* cell-free tests, the impact on ROS production in HepG2 cells, used as a model cell line, was evaluated. Different concentrations of OE ranging from 25 to 200 µg/mL were tested at 4 h of exposure (non-cytotoxic conditions). Exposure of HepG2 cells to tert-butyl hydroperoxide (TBH) resulted in a two-fold increase in ROS levels (Figure 6). However, pre-treatment with OE significantly attenuated TBH-induced ROS production, showing a concentration-dependent trend. The strongest reduction was observed at 200 and 100 µg/mL, whereas lower concentrations (50 and 25 µg/mL) produced a less pronounced effect. Interestingly, under the same experimental conditions, OE showed an ROS-reducing effect comparable to that of the positive control, NAC.

Research has shown that the polyphenolic content, including flavonoids and tannins, in hazelnut husks plays a crucial role in determining antioxidant activity. Del Rio et al. (2011) characterized the polyphenolic composition of hazelnut skin [19], while Göncüoğlu Taş et al. (2015) found that flavonoids accounted for nearly 60% of the total phenolic compounds in hazelnut skin [31]. These compounds are known to modulate cellular pathways that reduce oxidative stress and inflammation, thereby preventing tissue damage [34].

**Figure 7:**
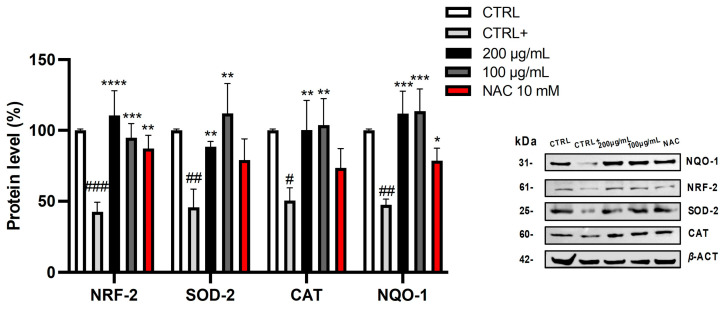
Effect of optimized hazelnut skin extract (200 and 100 μg/mL) on protein expression. Cell lysate was subjected to Western blot analysis and protein content was normalized against *β*-actin. Immunoreactive bands are from single experiment representative of multiple experiments and results from densitometric analysis are expressed as mean ± SD of three independent experiments (*n* = 3) and analyzed by one-way analysis of variance (ANOVA) followed by Tukey’s post hoc test; ^###^ *p* < 0.001, ^##^ *p* < 0.01, ad ^#^ *p* < 0.05 *vs*. control cells (CTRL); **** *p* < 0.0001, *** *p* < 0.001, ** *p* < 0.01, and * *p* < 0.05 *vs*. TBH-treated cells (CTRL+).

The obtained results are in agreement with observations from the non-cellular assays and with the findings reported by Rusu et al. (2019) [35]. In fact, they observed a protective effect of the hazelnut involucre extract from the oxidative stress induced by H_2_O_2_ in T47D-KBluc, A549, and HGF cell lines [35]. Although no previous studies have specifically investigated the ROS-inhibitory mechanism of hazelnut skin, the present research suggests that these effects are likely mediated by both direct scavenging activity (as shown in *in vitro* tests) and activation of the cellular antioxidant defense system. In particular, the induction of key enzymes such as superoxide dismutase (SOD), catalase (CAT), and NAD(P)H: quinone acceptor oxidoreductase 1 (NQO-1) appears to play a central role. As shown in Figure 7, TBH exposure led to a significant decrease in the protein expression of SOD-2, NRF-2, CAT, and NQO-1.

However, pre-treatment with OE or NAC restored or enhanced the basal levels of these proteins, further supporting the antioxidant potential of hazelnut skin. NRF-2 is a transcription factor that acts as a central regulator of the cellular antioxidant response. Under normal conditions, NRF-2 is kept in the cytoplasm by a protein called KEAP1. However, when cells are exposed to oxidative stress, NRF-2 translocates to the nucleus, where it binds to AREs (antioxidant response elements) in the DNA. This binding activates transcription of various antioxidant and cytoprotective genes, including those encoding SOD, CAT, NQO-1, and other enzymes involved in detoxifying ROS [36].

The observed effect is mechanism-based and not just chemical scavenging.

This study provides new insights into the mechanisms by which hazelnut skin contributes to reducing ROS levels and mitigating oxidative stress.

## 3. Discussion

The present study focused on optimizing the extraction process for hazelnut skins. Although it accounts for approximately 2.5% of the total kernel weight, this by-product is considered a significant waste product in the food industry, as it is removed during roasting due to its bitter taste. Consequently, there is an ongoing initiative to valorize it due to its potential as a source of bioactive compounds.

A 3^3^ full factorial experimental design was used to obtain an extract with the highest phenolic content and antioxidant activity. Five complementary *in vitro* tests were used to evaluate antioxidant activity, to study different mechanisms of action, and to obtain a more comprehensive assessment of the antioxidant profile.

There are interesting works on hazelnuts in the literature, each with a different extraction method and its own scientific peculiarities. For example, Esposito et al. [32] focus on hazelnut shells, using traditional methanolic extraction to isolate neolignans and diarylheptanoids, and evaluating them using DPPH, cytotoxicity assays, and apoptotic markers. Instead, Mencherini et al. [37] analyze roasted skin via hydroalcoholic PLE (30% EtOH, 125 °C, 1500 psi, 5 cycles; yield 33.2 g/100 g), with a predominantly applicative perspective. In the present study, the selected extraction method was ultrasound-assisted extraction, as it has been demonstrated to be the most appropriate for this by-product [38,39]. Further, other studies have performed an optimization process by comparing temperature, solvent type, and drug–solvent ratio or ultrasound power [38,40]. In this case, considering the results of previous research as a starting point, we decided to study the effects of time, temperature, and solvent on the extraction of hazelnut skin [35,39,41]. In this instance, the parameters used as dependent variables in process optimization were antioxidant assays, namely TPC, ABTS, DPPH, FRAP, and BCB. The developed models exhibit high predictive power, with the linear and quadratic ethanol effect governing the response surface geometry across all measured assays. Furthermore, the significance of the linear time term underscores the importance of optimizing process duration to prevent a loss of bioactivity. The multiple-optimization process is characterized by a high overall desirability index (0.96), reflecting the model’s high predictive power and enabling the identification of optimal extraction variables: an ethanol concentration of 21.13%, a temperature of 30 °C, and an extraction time of 70.86 min (1.181 h). These conditions confirm that maximum extraction efficiency for phenolic compounds and antioxidants is achieved by shifting the polarity of the system towards the aqueous phase, thus promoting the solubilization of highly polar phytochemicals that more concentrated hydroalcoholic mixtures would otherwise overlook. In particular, UHPLC-MS/MS analysis (Table 4) revealed that the main bioactive compound, the phenolic acid 3,5-dihydroxybenzoic acid, has a moderate polarity with a LogP of 0.934, making it optimally soluble in hydroalcoholic mixtures (~20% EtOH) rather than pure ethanol. This assumption is also confirmed by the literature demonstrating that a low concentration of ethanol (23%) was more effective for extracting polar poly-phenols, such as phenolic acids, than a high concentration of ethanol (85%) [42]. Similarly, it was seen that the extraction of catechins like gallogatechin, which have a comparable LogP to (-)-catechin (LogP of 0.877 and 0.938, respectively), increased with 20% of ethanol [43]. Furthermore, the optimization of temperature at the lower limit of the experimental range (30 °C) suggests a low energy input process that preserves the thermal integrity of bioactive molecules, avoiding the degradation phenomena often associated with prolonged contact times or excessive heat, as suggested by the negative coefficients observed for the time variable (C) in the TPC, ABTS, and FRAP responses. Hence, the present study differs in its conceptual and methodological framework, proposing an extraction process, significantly milder than high-temperature PLE (30% EtOH, 125 °C, 1500 psi, 5 cycles) [37], that allows for greater preservation of thermolabile metabolites. In addition, the use of hydroalcoholic mixtures with a low ethanol percentage, combined with ultrasound, represents a more sustainable strategy than traditional methanolic maceration [32], with better prospects for industrial transferability and greater adherence to the principles of green extraction.

With regard to the results obtained, it is evident that optimization has led to the successful extraction of a high content of phenolic compounds, as evidenced by the TPC result of 365.41 ± 8.14 mg GAE/g, which is approximately three times higher than that reported [32,38], and slightly higher than that in Mencherini et al. [37] (308.4 ± 4.6 mg GAE/g). It has been demonstrated that, at comparable temperatures, the duration of the extraction process is a key factor in optimizing the recovery of specialized metabolites from the matrix. For DPPH and ABTS, the results obtained are significantly superior to those reported by Özdemir et al. [38] and Pfeil et al. [44]. A comparison between the currently available data and those from FRAP and BCB was not possible.

Phytochemical profiling by UHPLC–MS/MS revealed a complex composition dominated by phenolic acids and flavonoids, with 3,5-dihydroxybenzoic acid as the most abundant compound. The prevalence of flavonoids such as catechin, epicatechin, procyanidin B2, naringenin, and luteolin provides a plausible chemical basis for the high antioxidant capacity observed [37,45]. These compounds are well known to act through complementary mechanisms, including radical scavenging and modulation of intracellular redox pathways, suggesting a synergistic contribution to the extract’s overall antioxidant effect. These compounds have already been reported in the literature in hazelnut skin extracts, both in the varieties analyzed and in other hazelnut varieties. The distinguishing factor pertains to the amount. The results obtained in this study were higher than those reported by Pfeil et al. [44], thereby highlighting the impact of cultivation location on final product quality. The findings were consistent with, or marginally lower than, those reported by Del Rio et al. [19], thereby highlighting the significance of aqueous solvents for the effective recovery of specialized metabolites from the matrix under consideration.

Moreover, some detected compounds could not be structurally assigned and were classified as unknown. These compounds may represent minor or previously unreported constituents and could contribute to the overall biological activity. Further studies employing advanced tools such as molecular networking or NMR would be required for their characterization.

The biological relevance of these findings was confirmed in HepG2 cells, where the optimized extract reduced intracellular ROS levels without inducing cytotoxicity at short exposure times. The ability of the extract to counteract TBH-induced oxidative stress, together with the restoration of key antioxidant proteins such as SOD-2, CAT, NQO-1, and NRF-2, suggests that OE exerts its antioxidant effects not only through direct radical scavenging but also by activating endogenous cellular defense mechanisms [23]. The involvement of the NRF-2 pathway further supports the role of hazelnut skin polyphenols as modulators of cellular redox homeostasis [29].

In conclusion, the results obtained in this study demonstrate that the optimized hazelnut skin extract does not merely exhibit antioxidant activity through direct radical scavenging but also modulates endogenous defense systems. This aspect is certainly of interest in the pharmacological and nutraceutical fields.

Overall, these results provide strong evidence that hazelnut skin, traditionally considered a food industry waste, represents a promising source of bioactive compounds. The optimized ultrasound-assisted extraction process enhances the recovery of bioactive compounds and supports the sustainable valorization of this agro-industrial residue within a circular economy framework.

## 4. Materials and Methods

### 4.1. Chemicals

HPLC-grade water (18 mΩ) was prepared by a Mill-Ω purification system (Millipore Corp., Bedford, MA, USA); analytical grade methanol (CAS No. 67-56-1), ethanol (CAS No. 64-17-5), formic acid (CAS No. 64-18-6), and dimethyl sulfoxide (DMSO, CAS No. 67-68-5)) were obtained from Merck (Darmstadt, Germany and Mollet del Vallés, Spain). Analytical standards were acquired from Sigma (St. Louis, MO, USA and Steinheim, Germany). All standards possessed a purity ≥ 98% (determined by HPLC). Folin–Ciocâlteu reagent (CAS No. 12111-13-6), sodium carbonate (CAS No. 497-19-8), 2,2-diphenyl-1-picrylhydrazyl (DPPH) (CAS No. 1898-66-4), 2,2′-azino-bis(3-ethylbenzothiazoline-6-sulfonic acid) (ABTS), potassium persulfate, 4,6-tris-(2-pyridyl)-*s*-triazine (CAS No. 3682-35-7), sodium acetate (CAS No. 127-09-3), *β*-carotene (CAS No. 7235-40-7), iron (III) chloride (FeCl_3_·6H_2_O) (CAS No. 10025-77-1), linoleic acid (CAS No. 60-33-3), potassium phosphate monobasic (CAS No. 7778-77-0), sodium chloride (CAS No. 7647-14-5), sodium hydroxide (NaOH, CAS No. 1310-73-2), sodium phosphate (CAS No. 7601-54-9), trizma hydrochloride (CAS No. 1185-53-1), 6-hydroxy-2,5,7,8-tetramethylchroman-2-carboxylic acid (Trolox) (CAS No. 53188-07-1), butylhydroxytoluene (BHT) (CAS No. 128-37-0), gallic acid (CAS No. 149-91-7), Tween 20 (CAS No. 9005-64-5), Dulbecco’s modified Eagle’s medium (DMEM, SID 56312060), Fetal Bovine Serum (FBS, MDL number: MFCD00132239), and penicillin–streptomycin (CID 131715954) were purchased from Sigma-Aldrich (Milan, Italy).

### 4.2. Plant Material

The skins represent the thin cuticle that covers every single hazelnut, which is removed for its bitter taste. The present study utilized a mixture of different cultivars, namely *Nocchione*, *Gentile Romana*, and *Tonda di Giffoni*, cultivated in Basilicata and in the Viterbo area, and obtained from hazelnuts harvested in September 2021. The plant material was stored in a dark and dry place until extraction. Prior to this step, it was made uniform in terms of size, by grinding it into fragments of approximately 3–4 mm using a mortar and pestle, to increase the surface area of contact with the solvent and thus enhance the efficiency of the extraction process.

### 4.3. Optimization of Extraction Procedure and Extraction Yield

The extraction procedure was carried out using an ultrasonic bath (Branson 1800 Ultrasonic (Emerson, St. Louis, MO, USA) bath with a frequency and amplitude of 40 kHz and 100%, respectively) and a thermo-heating probe (ISCO GTR 190, ISCO, Louisville, KY, USA) to keep the temperature constant during the process. Ultrasound-assisted extraction was chosen for its ability to recover specialized metabolites from plant species, particularly hazelnut skin, as previously reported [38,41]. Ultrasound power and frequency were kept constant during the experiments to minimize variability and to focus the optimization on solvent composition, temperature, and extraction time.

The 3^3^ Full Factorial Design (FFD) was employed to optimize extraction parameters. The independent variables were determined as temperature (30–50–70 °C), time (1–2–3 h), and solvent (100% EtOH–50% EtOH–0% EtOH), keeping constant and equal to 1:100 the ratio between the drug and solvent. This ratio was chosen based on previous in-house experiments. Different antioxidant assays were used as dependent variables to analyze the response in terms of antioxidant activity. The real and coded values of independent variables for each factor are listed in Table 5.

Analysis of variance (ANOVA) was used to evaluate the impact of the selected independent variables, both individually and in combination, on the dependent variables (Total Phenolic Content, as determined by the TPC assay, and antioxidant activity, as evaluated by the ABTS, DPPH, BCB, and FRAP assays) of hazelnut skin extract. The experimental data were modeled using the subsequent second-order polynomial equation (Equation (1)) [16,17,18]:

(1) $$Y\;=\;\beta_{0}+\sum_{i=1}^{3}\beta_{i}X_{1}+\sum_{i=1}^{3}\beta_{ii}X_{i}^{2}+\sum_{i=1}^{3}\beta_{iJ}X_{i}X_{j}\;+\;\beta_{123}X_{1}X_{2}X_{2}$$

where Y represents the dependent variables; $\beta_{0}$ is the intercept, i.e., the predicted value of the response when all variables are at their central level (0 in coded values); $\beta_{i}X_{1}$ is linear effects (A, B, C), representing the average variation in response to a change in a single variable independently of the others; $\beta_{ii}X_{i}^{2}$ is the quadratic effects (A^2^, B^2^, C^2^), identifying the curvature of the model; $\beta_{iJ}X_{i}X_{j}$ and $\beta_{123}X_{1}X_{2}X_{2}$ are the interactions (AB, AC, BC, ABC), describing how the effect of one variable changes based on the level of another.

ANOVA and the multiple optimization of the model were performed using DesignExpert13 (version 13, Design-Expert Software, Stat-Ease Inc., Minneapolis, MN, USA).

After extraction, the 27 extracts were centrifuged and filtered twice. The samples thus obtained were dried using a rotary evaporator at 37 °C. The dried extracts were stored at room temperature in the dark until they were used for subsequent analysis. For each extract, the extraction yields were calculated according to the following formula [16]:

(2) $$\%=\frac{dried\;extract\;(g)}{dried\;hazelnut\;skin\;(g)}\times 100$$

### 4.4. Total Phenolic Content (TPC)

The Total Content of Phenolic compounds was determined using Folin–Ciocâlteu reagent. A total of 75 µL of the sample, or solvent for the blank, was mixed with 500 µL of Folin-Ciocâlteu reagent and 500 µL of a 10% (*w*/*v*) sodium carbonate (Na_2_CO_3_) aqueous solution. In addition, 425 µL of water was added to achieve a final volume of 1.5 mL. The mixture was shaken and incubated for one hour in the dark at room temperature before evaluating its absorbance at 723 nm by employing a UV–VIS spectrophotometer (SPECTROstar^Nano^ BMG Labtech, Ortenberg, Germany). The experiment was performed in triplicate and the results were expressed as milligrams of gallic acid equivalents per gram of dried extract (mg GAE/g) ± standard deviation (SD) [46].

### 4.5. 2,2′-Azino-Bis(3-Ethylbenzothiazoline-6-Sulfonic Acid) (ABTS) Assay

ABTS^•+^ is a cationic radical generated by reacting 7.7 mM ammonium salt of 2,2′-azino-bis(3-ethylbenzothiazoline-6-sulfonic acid) (ABTS) aqueous solution with 2.9 mM potassium persulfate (K_2_S_2_O_8_) aqueous solution in a 1:1 ratio. The mixture was incubated in the dark at room temperature for 12–16 h. Before use, the ABTS^•+^ solution was diluted with methanol to obtain an absorbance of 1.1 ± 0.02 at 734 nm. For the assay, 235 μL of the ABTS solution was added to 15 μL of the sample, or solvent for the blank. The absorbance was measured at 734 nm after 120 min of incubation in the dark at room temperature. For each sample, three replicates were performed. The results were expressed as milligrams of Trolox equivalents per gram of dried extract (mg TE/g) ± SD [47].

### 4.6. 2,2-Diphenyl-1-Picrylhydrazyl (DPPH) Assay

The 2,2-diphenyl-1-picrylhydrazyl (DPPH) is a stable free radical and its scavenging is used to evaluate the antioxidant capacity of samples. A total of 200 μL of the methanolic DPPH solution (200 µM) was added to 50 μL of the sample, or solvent for the blank. The absorbance was measured at 515 nm after 30 min of incubation in the dark at room temperature. Data represent the mean values of three independent replicates ± SD and were expressed as milligrams of Trolox equivalents per gram of dried extract (mg TE/g) [48].

### 4.7. Ferric Reducing Antioxidant Power (FRAP) Assay

The FRAP reagent was obtained by mixing three solutions in a ratio 10:1:1. The first solution consists of a 300 mM acetate buffer (pH 3.6), the second solution was obtained by dissolving 4,6-tris-(2-pyridyl)-*s*-triazine (TPTZ) in HCl 40 mM to obtain a 10 mM solution, while the third consisted of an aqueous solution of iron chloride hexahydrate (FeCl_3_·6H_2_O) at a concentration of 20 mM. Each sample (25 µL) or solvent for the blank was mixed with 225 µL of the complex solution and incubated in the dark at 37 °C for 40 min. The absorbance was measured at 593 nm. For each sample, three replicates were performed. The results were expressed as milligrams of Trolox equivalents per gram of dried extract (mg TE/g) ± SD [49].

### 4.8. β-Carotene Bleaching (BCB) Assay

The *β*-Carotene Bleaching assay evaluates the “*whitening*” of *β*-carotene in the presence of an antioxidant sample. Preparation of an emulsion is required for the assay as follows: 0.6 mg of *β*-Carotene dissolved in 0.6 mL of chloroform (CHCl_3_), 200 mg of Tween 20, and 45 µL of linoleic acid. Subsequently, chloroform was removed using a rotary evaporator at room temperature and 50 mL of oxygenated distilled water was added. A total of 950 μL of the emulsion was mixed with 50 μL of the sample at a concentration of 2 mg/mL, or solvent for the blank. Butylhydroxytoluene (BHT) was used as a positive control. The absorbance was measured immediately (T_0′_) and after 180 min (T_180′_) of incubation at 50 °C. The results are expressed as a percentage of antioxidant activity (% AA) and calculated with the following equation:

(3) $$\%AA=\left(1-\frac{Abs\;sample\;T0^{\prime }-Abs\;sample\;T180^{\prime }}{Abs\;blank\;T0^{\prime }-Abs\;blank\;T180^{\prime }}\right)\times 100$$

where *Abs sample T*_0′_ and *Abs blank T*_0′_ are the absorbance of the extract and the blank before incubation and *Abs sample T*_180′_ and *Abs blank T*_180′_ are the absorbance after 180 min of incubation of the extract and the blank, respectively [50].

### 4.9. Phytochemical Profile Analysis by UHPLC-MS

OE was analyzed using the UHPLC-DAD-ESI-Orbitrap Exploris^TM^ 120 Mass Spectrometer (Thermo Fisher Scientific, Milan, Italy) with a reversed-phase system (Vanquish UHPLC Thermo Scientific system coupled to DAD), equipped with a binary pump. A Luna Omega PS C18 produced by Phenomenex (100 × 2.1 mm, 1.6 μm, 100 Å) was used as a column.

As mobile phases, water + 0.1% (*v*/*v*) formic acid (A) and MeOH + 0.1% (*v*/*v*) formic acid (B) were employed and programmed in the following gradient: 0–5 min 5–35% B, 5–25 min 35–100% B; 25–35 min 100% B. Before use, mobile phases were filtered through a 0.22 µm membrane. The total analysis time was 35 min at a column temperature of 30 °C and a sample compartment temperature of 8 °C. The injected volume was 5 µL with a constant flow rate of 0.2 mL/min.

Electrospray mass spectra data were acquired in negative ionization mode with a voltage set at 3500 V, maintaining a resolution of 120,000 both in Full MS and dd-MS^2^ scans and across an *m*/*z* range of 80–1200. Nitrogen was used as a collision gas and the collision-induced dissociation (CID) of the analytes was achieved using 30 eV. Ion transfer tube and vaporizer temperatures were 300 °C and 280 °C, respectively. Sheath gas, aux gas, and sweep gas were 40, 20, and 0, respectively.

EASY-IC^TM^ was used for internal mass calibration. The full MS/dd-MS^2^ quantification method was developed for standard compounds and quantification of the data was carried out using Chromeleon 7.3.1 Software (Thermo Scientific^TM^ Dionex^TM^) [51].

The quantification of identified compounds reported was performed using external calibration curves prepared with the corresponding available authentic reference standards. In particular, standard 3,5-dihydroxybenzoic acid, catechin, epicatechin, luteolin, malic acid, naringenin, phloridzin dihydrate, procyanidin B2, quinic acid and ursolic acid were solubilized in 80% MeOH between 0.1 and 100 ppm. The analytical response was evaluated within the selected concentration ranges, and the quantitative results should be interpreted as referring to the compounds for which standard-based calibration was available.

MS/MS spectra were interpreted by comparison with literature-reported fragmentation patterns and spectral databases (e.g., MassBank and PubChem), focusing on diagnostic ions and characteristic neutral losses typical of phenolic compounds.

### 4.10. Cell Culture Conditions

The human hepatocellular carcinoma (HepG2) cells, used as a cellular model [52], were purchased from ATCC (Manassas, VA, USA) (HB-8065). Cells were grown in DMEM supplemented with 10% FBS, streptomycin (100 μg/mL), penicillin (100 units/mL) and 2 mM glutamine in a 5% CO_2_ humidified incubator at 37 °C.

### 4.11. Cell Viability

The effect of OE on cell viability was evaluated via the MTT assay. HepG2 cells were seeded in a 96-well plate (1.5 × 10^4^ cells/well), and treated for 4 h and 24 h with different concentrations (25–200 µg/mL) of OE, followed by incubation with MTT solution (0.75 mg/mL) for 4 h. The formazan crystals produced by viable cells were dissolved in a mixture of DMSO/isopropanol (1:1). A UV–Vis spectrophotometer (SPECTROstar^Nano^ BMG Labtech, Ortenberg, Germany) was used for absorbance quantification at 560 nm [53].

### 4.12. Measurement of Intracellular ROS

The protective effect of OE in reducing ROS production was quantified with DCFH-DA, as previously described [54].

HepG2 cells were seeded in a 24-well plate (1 × 10^5^ cells/well), pre-treated with different concentrations of OE (200–25 µg/mL) or *N*-acetyl-L-cysteine (NAC, 10 mM), used as a positive control, for 4 h and then incubated with TBH (5 mM) for 1 h. Subsequently, DCFH-DA (10 µM, at 37 °C) was added to the wells for 30 min, and fluorescence was measured by using a BD FACSCanto II flow cytometer (BD Pharmingen, San Jose, CA, USA) (*λ*_ex_ 485 nm and *λ*_em_ 515–540 nm).

### 4.13. Western Blot Analysis

HepG2 cells were seeded in a 12-well plate (2 × 10^5^ cells/well) and treated with two concentrations (200 and 100 µg/mL) of OE or NAC (10 mM) for 4 h. Changes in intracellular protein levels were evaluated in the presence of the stressor (5 mM for 1 h) via Western blotting. Cell lysates were prepared using RIPA buffer, phosphatase and protease inhibitors. Protein content was quantified in the cell supernatant by using the Bradford assay. Equal amounts of proteins were loaded and separated into 12% SDS-PAGE gels (*w*/*v*) and transferred to nitrocellulose membranes. Then, membranes were incubated overnight with the primary antibody at 4 °C. Anti-*β*-actin (1:5000), anti-SOD2 (1:1000), anti-CAT (1:1000), and anti-NRF-2 (1:1000) were purchased from Thermo Fisher Scientific, Milan, Italy, and anti-NQO1 (1:100) antibody was purchased from Santa Cruz Biotechnology, Inc., Santa Cruz, CA, USA. The primary antibodies were captured with suitable peroxidase-conjugated secondary antibody at room temperature for 1 h. The immunoreaction bands were visualized using the iBright 1500 Imaging system (Thermo Fisher Scientific, Milan, Italy). Densitometric analysis was performed by using ImageJ software (version 1.53k, National Institutes of Health, Bethesda, MD, USA) and the results were expressed as a percentage of the value of the untreated control sample (100%) [46].

### 4.14. Statistical Analysis

All experiments were performed in triplicate and data were expressed as the mean ± standard deviation. Statistical analysis was carried out using GraphPad Prism 5 Software, Inc. (San Diego, CA, USA). To determine the differences among the samples, one-way ANOVA tests were performed with the Tukey–Kramer post hoc test. The level of significance was set at *p*-values ≤ 0.05.

## 5. Conclusions

This study demonstrates that hazelnut (*Corylus avellana* L.) skin, a widely generated by-product of the food industry, represents a sustainable source of bioactive compounds with significant antioxidant potential. By applying the 3^3^ FFD, optimal ultrasound-assisted extraction conditions were successfully identified: 30 °C, 70.86 min (1.181 h), and 21.13% ethanol. The optimized extraction process yielded a phenolic-rich extract, characterized by a high abundance of flavonoids as confirmed by UHPLC–MS/MS analysis. In addition, it exhibited strong antioxidant capacity, as evaluated by different *in vitro* assays (TPC, ABTS, DPPH, FRAP, and BCB), which were used as response variables during the optimization process.

Biological evaluation of HepG2 cells revealed that the optimized extract exerts a significant antioxidant effect, effectively reducing intracellular ROS levels under oxidative stress. This activity is associated with the activation of endogenous antioxidant defense pathways, as demonstrated by the upregulation of key antioxidant markers, including SOD-2, CAT, NRF-2, and NQO-1.

The results obtained provide new insights into the antioxidant mechanisms of hazelnut skin extracts and support their valorization as a new raw material. Overall, the combination of optimized extraction parameters, well-characterized phytochemical composition, and demonstrated cellular antioxidant activity suggests that hazelnut skin represents a promising candidate as a functional ingredient with potential application in pharmaceutical and nutraceutical fields, contributing to advancing circular economy strategies in the agri-food sector.

## Figures and Tables

**Table 1:** Independent variables used for FFD and results of antioxidant activity: Total Phenolic Content (TPC), 2,2′-azino-bis(3-ethylbenzothiazoline-6-sulfonic acid) (ABTS), 2,2-diphenyl-1-picrylhydrazyl (DPPH), Ferric Reducing Antioxidant Power (FRAP), and *β*-Carotene Bleaching assay (BCB).

	Independent Variables			Dependent Variables				
Run	A	B	C	TPC	ABTS	DPPH	FRAP	BCB
	(% EtOH)	(°C)	(h)	mg GAE/g ^1^	mg TE/g ^2^	mg TE/g ^2^	mg TE/g ^2^	% AA at 2 mg/mL ^3^
**1**	100 (+1)	30 (−1)	1 (−1)	177.88 ± 10.92 ^d^	844.23 ± 15.10 ^g^	1951.90 ± 91.05 ^d,e^	1371.14 ± 134.34 ^c^	1.13 ± 0.23 ^o^
**2**	100 (+1)	30 (−1)	2 (0)	149.61 ± 1.84 ^d,e^	644.22 ± 43.06 ^h,i^	1802.43 ± 75.90 ^d,e,f^	1145.24 ± 90.62 ^c^	4.71 ± 0.12 ^o^
**3**	100 (+1)	30 (−1)	3 (+1)	122.78 ± 1.89 ^e,f^	511.40 ± 39.88 ^i,j^	1332.40 ± 132.62 ^e,f^	981.86 ± 77.71 ^c,d^	7.26 ± 0.47 ^n,o^
**4**	50 (0)	30 (−1)	1 (−1)	379.46 ± 15.80 ^a^	1772.54 ± 62.61 ^a,b,c,d^	4298.96 ± 331.61 ^a,b,c^	2831.25 ± 171.97 ^a,b^	52.06 ± 1.72 ^g,h^
**5**	50 (0)	30 (−1)	2 (0)	382.49 ± 12.46 ^a^	1799.72 ± 52.43 ^a,b,c^	4578.43 ± 377.51 ^a,b^	3048.14 ± 181.53 ^a^	73.09 ± 2.27 ^b,c,d^
**6**	50 (0)	30 (−1)	3 (+1)	342.18 ± 14.50 ^a,b,c^	1489.73 ± 42.67 ^f^	4004.60 ± 303.44 ^a,b,c^	2857.70 ± 143.28 ^a,b^	66.37 ± 6.51 ^c,d,e,f^
**7**	0 (−1)	30 (−1)	1 (−1)	347.03 ± 24.72 ^a,b,c^	1852.37 ± 53.54 ^a,b^	4279.49 ± 182.30 ^a,b,c^	2776.19 ± 110.13 ^a,b^	87.89 ± 2.09 ^a^
**8**	0 (−1)	30 (−1)	2 (0)	316.52 ± 19.26 ^c^	1769.14 ± 67.62 ^a,b,c,d^	3870.59 ± 198.52 ^b,c^	2503.28 ± 70.62 ^b^	65.24 ± 2.40 ^d,e,f^
**9**	0 (−1)	30 (−1)	3 (+1)	324.37 ± 11.81 ^b,c^	1651.94 ± 35.83 ^c,d,e,f^	3915.26 ± 162.37 ^b,c^	2460.24 ± 231.03 ^b^	73.98 ± 0.57 ^b,c^
**10**	100 (+1)	50 (0)	1 (−1)	137.83 ± 10.55 ^d,e,f^	673.95 ± 27.16 ^g,h,i^	1565.33 ± 52.90 ^d,e,f^	1154.86 ± 39.74 ^c^	4.71 ± 0.13 ^o^
**11**	100 (+1)	50 (0)	2 (0)	149.88 ± 6.20 ^d,e^	682.87 ± 28.81 ^g,h,i^	1726.26 ± 58.14 ^d,e,f^	1185.76 ± 71.34 ^c^	13.84 ± 1.17 ^m,n^
**12**	100 (+1)	50 (0)	3 (+1)	96.62 ± 5.76 ^f^	424.77 ± 38.87 ^j^	1126.23 ± 80.66 ^f^	679.50 ± 52.23 ^d^	6.35 ± 0.45 ^n,o^
**13**	50 (0)	50 (0)	1 (−1)	348.95 ± 7.82 ^a,b,c^	1722.43 ± 29.02 ^a,b,c,d^	4179.84 ± 418.27 ^a,b,c^	3103.92 ± 65.90 ^a^	32.10 ± 0.98 ^k,l^
**14**	50 (0)	50 (0)	2 (0)	315.77 ± 12.63 ^c^	1511.81 ± 58.66 ^e,f^	3745.74 ± 236.60 ^c^	2462.17 ± 95.07 ^b^	38.69 ± 3.44 ^j,k^
**15**	50 (0)	50 (0)	3 (+1)	342.00 ± 25.37 ^a,b,c^	1680.82 ± 43.00 ^b,c,d,e,f^	4135.17 ± 169.83 ^a,b,c^	2724.74 ± 154.70 ^a,b^	48.41 ± 4.58 ^h,i^
**16**	0 (−1)	50 (0)	1 (−1)	387.35 ± 13.50 ^a^	1862.56 ± 95.17 ^a,b^	4295.52 ± 228.67 ^a,b,c^	2701.89 ± 176.94 ^a,b^	63.79 ± 2.37 ^e,f^
**17**	0 (−1)	50 (0)	2 (0)	348.63 ± 25.31 ^a,b,c^	1805.66 ± 91.48 ^a,b,c^	4397.46 ± 261.28 ^a,b,c^	2648.99 ± 155.94 ^a,b^	70.55 ± 2.46 ^b,c,d,e^
**18**	0 (−1)	50 (0)	3 (+1)	343.46 ± 16.69 ^a,b,c^	1696.05 ± 140.95 ^a,b,c,d,e^	4240.54 ± 212.48 ^a,b,c^	2755.75 ± 175.49 ^a,b^	76.28 ± 6.19 ^b^
**19**	100 (+1)	70 (+1)	1 (−1)	151.39 ± 10.43 ^d,e^	776.29 ± 29.65 ^g,h^	2082.47 ± 170.02 ^d^	1269.19 ± 79.54 ^c^	25.09 ± 1.59 ^l^
**20**	100 (+1)	70 (+1)	2 (0)	146.13 ± 6.72 ^d,e,f^	723.63 ± 54.68 ^g,h^	2012.03 ± 158.56 ^d,e^	1177.71 ± 92.50 ^c^	27.88 ± 2.26 ^l^
**21**	100 (+1)	70 (+1)	3 (+1)	126.15 ± 6.74 ^d,e,f^	608.13 ± 38.39 ^h,i,j^	1588.24 ± 44.64 ^d,e,f^	991.48 ± 97.92 ^c,d^	15.99 ± 0.87 ^m^
**22**	50 (0)	70 (+1)	1 (−1)	339.14 ± 23.04 ^a,b,c^	1764.90 ± 92.72 ^a,b,c,d^	4289.80 ± 225.84 ^a,b,c^	2816.10 ± 126.72 ^a,b^	42.45 ± 2.32 ^i,j^
**23**	50 (0)	70 (+1)	2 (0)	363.94 ± 18.37 ^a,b,c^	1878.70 ± 41.48 ^a,b^	4666.63 ± 412.19 ^a^	2999.32 ± 277.81 ^a^	48.61 ± 3.91 ^h,i^
**24**	50 (0)	70 (+1)	3 (+1)	343.07 ± 32.82 ^a,b,c^	1734.32 ± 57.46 ^a,b,c,d^	4358.52 ± 247.44 ^a,b,c^	2662.94 ± 236.10 ^a,b^	55.20 ± 2.54 ^g,h^
**25**	0 (−1)	70 (+1)	1 (−1)	371.83 ± 11.60 ^a,b^	1891.19 ± 15.57 ^a^	4522.31 ± 270.66 ^a,b^	2711.75 ± 179.35 ^a,b^	50.46 ± 3.05 ^g,h,i^
**26**	0 (−1)	70 (+1)	2 (0)	314.27 ± 10.90 ^c^	1574.66 ± 103.65 ^d,e,f^	4154.64 ± 278.01 ^a,b,c^	2475.63 ± 155.39 ^b^	58.03 ± 0.93 ^f,g^
**27**	0 (−1)	70 (+1)	3 (+1)	357.91 ± 28.41 ^a,b,c^	1736.62 ± 85.47 ^a,b,c,d^	4440.99 ± 280.34 ^a,b,c^	2724.01 ± 139.23 ^a,b^	69.60 ± 0.64 ^b,c,d,e^

Results are expressed as mean ± standard deviation of mg/g of dried extract. In each column, significant differences (*p* < 0.05) are highlighted with different letters (a–o). ^1^ mg GAE/g = mg of Gallic Acid Equivalents per gram of dried extract; ^2^ mg TE/g = mg of Trolox Equivalents per gram of dried extract; ^3^ % AA = percentage of antioxidant activity at 2 mg/mL.

**Table 2:** Second-degree polynomial equation and statistical analysis.

	Intercept	A	B	C	AB	AC	BC	A ^2^	B ^2^	C ^2^	ABC
**TPC ^1^**	342.668	−102.95	−1.58278	−13.4622	−6.89083	−3.42333	6.65083	−107.403	7.01542	4.22375	2.64
***p*** **-values**		<0.0001 *	0.7447	0.0112 *	0.2546	0.5663	0.271	<0.0001 *	0.3773	0.5925	0.7173
**ABTS ^2^**	1710.68	−552.817	19.6194	−90.3711	14.9317	−19.055	38.5633	−513.804	35.204	−19.711	14.8512
***p*** **-values**		<0.0001 *	0.3912	0.0007 *	0.5919	0.495	0.1752	<0.0001 *	0.3434	0.5927	0.6629
**DPPH ^3^**	4206.28	−1273.86	115.643	−129.093	−38.0492	−87.6917	64.2717	−1302.55	171.716	−85.3037	−19.705
***p*** **-values**		<0.0001 *	0.0378 *	0.0221 *	0.5556	0.1828	0.3236	<0.0001 *	0.0548	0.322	0.8025
**FRAP ^4^**	2860.51	−766.722	−8.16167	−105.448	−19.295	−74.3767	21.6808	−992.196	22.5877	−15.5223	−27.08
***p*** **-values**		<0.0001 *	0.8374	0.0146 *	0.6924	0.1381	0.6569	<0.0001 *	0.7262	0.8097	0.6506
**BCB ^5^**	53.8635	−28.27	−2.13444	3.32	8.74	−1.5875	1.355	−13.4277	3.61563	−4.03771	−6.035
***p*** **-values**		<0.0001 *	0.3439	0.1476	0.0043 *	0.5625	0.6207	0.0013 *	0.323	0.2712	0.0831

Bold text indicates the different methodological categories considered: ^1^ Total Phenolic Content (TPC), ^2^ 2,2′-azino-bis(3-ethylbenzothiazoline-6-sulfonic acid) (ABTS), ^3^ 2,2-diphenyl-1-picrylhydrazyl (DPPH), ^4^ Ferric Reducing Antioxidant Power (FRAP), and ^5^ *β*-Carotene Bleaching assay (BCB). A: % EtOH/H_2_O; B: temperature; C: time. * Variables with a *p*-value < 0.05 are significant.

**Table 3:** Comparison between the predicted and obtained results for the optimized extract in the *in vitro* antioxidant assays.

Assays	Predicted Results (95% CI)	Obtained Results *
TPC (mg GAE/g ^1^)	363.547–411.153	365.41 ± 8.14
ABTS (mg TE/g ^2^)	1847.96–2070.22	1929.76 ± 117.38
DPPH (mg TE/g ^3^)	4353.29–4867.99	3936.95 ± 143.94
FRAP (mg TE/g ^4^)	2868.38–3258.29	2911.90 ± 165.32
BCB (% AA ^5^)	63.37–85.22	74.90 ± 2.25

* Data are expressed as the mean ± SD from three experiments (*n* = 3); ^1^ Total Phenolic Content (TPC); ^2^ 2,2′-azino-bis(3-ethylbenzothiazoline-6-sulfonic acid) (ABTS); ^3^ 2,2-diphenyl-1-picrylhydrazyl (DPPH); ^4^ Ferric Reducing Antioxidant Power (FRAP); and ^5^ *β*-Carotene Bleaching assay (BCB).

**Table 4:** Detected compounds in the optimized hazelnut skin extract using UHPLC-MS/MS.

Pk. No.	RT (min)	[M-H]^−^ (*m*/*z*) Calculated	[M-H]^−^ (*m*/*z*) Observed	Predicted Molecular Formula	MS/MS (*m*/*z*)	Compound Identity	Amount (mg/g)
1	1.51	341.1078	341.1088	C_12_H_22_O_11_	179, 161, 143, 119, 113, 101, 89 (100)	Caffeoyl hexoside	
2	1.87	133.0143	133.0144	C_4_H_6_O_5_	115 (100)	Malic acid	1.350 ± 0.017
3	7.13	153.0182	153.0194	C_7_H_6_O_4_	109 (100)	3,5-dihydroxybenzoic acid	2.711 ± 0.084
4	8.20	577.1341	577.1357	C_30_H_26_O_12_	245, 203, 201, 125	Procyanidin-*type*	
5	8.83	577.1341	577.1354	C_30_H_26_O_12_	245, 203, 201, 125	Procyanidin-*type*	
6	8.91	289.0707	289.0718	C_15_H_14_O_6_	245, 203, 151, 125, 123, 109 (100)	+/− Catechin	1.027 ± 0.033
7	9.20	577.1341	577.1354	C_30_H_26_O_12_	407, 289 (100), 245, 203, 201, 161, 125	Procyanidin B2	0.099 ± 0.007
8	10.11	577.1341	577.1354	C_30_H_26_O_12_	245, 203, 201, 125	Procyanidin-*type*	
9	10.20	289.0707	289.0718	C_15_H_14_O_6_	245, 203, 151, 125, 123, 109 (100)	Epicatechin	0.161 ± 0.014
10	13.27	343.1176	343.1187	C_19_H_20_O_6_	203 (100), 201	Diarylheptanoids (carpinontriol B)	
11	13.99	435.1286	435.1296	C_21_H_24_O_10_	273, 167 (100), 123	Phloridzin dihydrate	0.160 ± 0.002
12	14.35	187.0965	187.0976	C_9_H_16_O_4_	125 (100)	Azelaic acid	
13	16.38	431.0973	431.0984	C_21_H_20_O_10_	285 (100), 255, 227	Kaempferol rhamnoside	
14	16.52	271.0601	271.0612	C_15_H_12_O_5_	151 (100)	Naringenin	0.030 ± 0.0002
15	17.57	285.0394	285.0404	C_15_H_10_O_6_	203 (100)	Luteolin	0.344 ± 0.016
16	18.10	225.1485	225.1496	C_13_H_22_O_3_	203 (100), 201	Unknown	
17	19.93	207.1380	207.1389	C_13_H_20_O_2_	203	Unknown	
18	20.62	293.1747	293.1758	C_17_H_26_O_4_	249, 236, 221, 193 (100)	Unknown	
19	23.88	315.2530	315.2540	C_18_H_36_O_4_	297, 279, 269, 203 (100), 201	Dihydroxyoctadecanoic acid	
20	24.39	315.2530	315.2540	C_18_H_36_O_4_	297, 279, 269, 203 (100), 201	Dihydroxyoctadecanoic acid	
21	27.60	249.1485	249.1496	C_15_H_22_O_3_	205 (100), 203, 112	Unknown	
22	28.01	455.352	455.3531	C_30_H_48_O_3_	203 (100)	Ursolic acid	0.112 ± 0.004
23	28.10	279.2319	279.2329	C_18_H_32_O_2_	261, 203 (100)	Linoleic acid	
24	28.63	255.2319	255.2329	C_16_H_32_O_2_	203 (100), 201	Palmitic acid	
25	28.90	281.2475	281.2485	C_18_H_34_O_2_	255, 203 (100), 179	Fatty acids	

Results are expressed as mean ± standard deviation of mg/g of dried extract.

**Table 5:** Coded level for independent variables.

	Symbols	Coded Level		
Variables		−1	0	1
% EtOH	A	0	50	100
Temperature (°C)	B	30	50	70
Time (h)	C	1	2	3

## Data Availability

The original contributions presented in this study are included in the article/Supplementary Materials. Further inquiries can be directed to the corresponding authors.

## Supplementary Materials

The following supporting information can be downloaded at https://www.mdpi.com/article/10.3390/ph19040539/s1, Table S1: Matrix used for the 33 full factorial experimental design; Table S2: ANOVA analysis of the model; Table S3: Fit statistics of the model; Figure S1: Normal Plot of residuals for Total Phenolic Content (TPC), 2,2′-azino-bis(3-ethylbenzothiazoline-6-sulfonic acid) (ABTS), 2,2-diphenyl-1-picrylhydrazyl (DPPH), Ferric Reducing Antioxidant Power (FRAP), and *β*-Carotene Bleaching assay (BCB).

## Abbreviations

The following abbreviations are used in this manuscript:

ABTS	2,2′-Azino-bis(3-ethylbenzothiazoline-6-sulfonic acid)
BCB	*β*-Carotene Bleaching
DPPH	2,2-Diphenyl-1-picrylhydrazyl
FRAP	Ferric Reducing Antioxidant Power
FFD	Full Factorial Design
MTT	3-(4,5-Dimethylthiazol-2-yl)-2,5-diphenyltetrazolium bromide
OE	Optimized hazelnut skin extract
TPC	Total Phenolic Content
