# Supplementary material for: Technological Optimization and Antioxidant Efficacy via the NRF-2-Mediated Defense Pathway of Corylus avellana L. Skin Extracts: A Sustainable Approach for Developing Health-Promoting Natural Products
Source: Pharmaceuticals (Basel). 2026 Mar 27;19(4):539. doi: 10.3390/ph19040539 (PMC13118632; doi:10.3390/ph19040539)
Supplement: Supplementary file 1 [file pharmaceuticals-19-00539-s001.zip › pharmaceuticals-4204141-supplementary.pdf]

pharmaceuticals

Supplementary material

Technological Optimization and Antioxidant Efficacy via the NRF-2-Mediated Defense Pathway of *Corylus avellana* L. Skin Extracts: A Sustainable Approach for Developing Health-Promoting Natural Products

Immacolata Faraone [1,†], Maria Ponticelli [1,†], Claudia Mangieri [2], Ilaria Nigro [1], Ludovica Lela [1,*], Antonio Vassallo [1,*], Carlo Cosentino [3], Nikolay T. Tzvetkov [4,5], Vittorio Carlucci [1], Maria Francesca Armentano [1,‡] and Luigi Milella [1,‡]

1 Department of Health Sciences, University of Basilicata, Via dell'Ateneo Lucano 10, 85100 Potenza, Italy; immacolata.faraone@unibas.it (I.F.); maria.ponticelli@unibas.it (M.P.); ilaria.nigro@unibas.it (I.N.); vittorio.carlucci@unibas.it (V.C.); mariafrancesca.armentano@unibas.it (M.F.A.); luigi.milella@unibas.it (L.M.)

2 Department of Basic and Applied Sciences, University of Basilicata, Via dell'Ateneo Lucano 10, 85100 Potenza, Italy; claudia.mangieri@unibas.it

3 Department of Agriculture, Forest, Food and Environmental Sciences, University of Basilicata, Via dell'Ateneo Lucano 10, 85100 Potenza, Italy; carlo.cosentino@unibas.it

4 Department of Biochemical Pharmacology & Drug Design, Institute of Molecular Biology "Roumen Tsanev", Bulgarian Academy of Sciences, Acad. G. Bonchev Str., Bl. 21, 1113 Sofia, Bulgaria; ntzvetkov@gmx.de

5 Institute of Genetics and Animal Biotechnology, Polish Academy of Sciences, 05-552 Magdalenka, Poland

* Correspondence: ludovica.lela@unibas.it (L.L.); antonio.vassallo@unibas.it (A.V.); Tel.: +39-0971-205-624 (A.V.)

† These authors contributed equally to this work.

‡ These authors contributed equally to this work.

Index

Table S1. Matrix used for the 33 full factorial experimental design.

Table S2. ANOVA analysis of the model.

Table S3. Fit statistics of the model.

Figure S1. Normal Plot of residuals for Total Phenolic Content (TPC), 2,2′-azino-bis(3-ethylbenzothiazoline-6-sulfonic acid) (ABTS), 2,2-diphenyl-1-picrylhydrazyl (DPPH), Ferric Reducing Antioxidant Power (FRAP), and *β*-Carotene Bleaching assay (BCB).

Table S1. Matrix used for the 3^3 full factorial experimental design.

Run	Independent Variables			Dependent Variables				
	X_1 (% EtOH)	X_2 (°C)	X_3 (h)	TPC mg GAE/g[1]	ABTS mg TE/g[2]	DPPH mg TE/g[2]	FRAP mg TE/g[2]	BCB % AA at 2 mg/mL[3]
**1**	100	30	1	177.88	844.23	1951.90	1371.14	1.13
**2**	100	30	2	149.61	644.22	1802.43	1145.24	4.71
**3**	100	30	3	122.78	511.40	1332.40	981.86	7.26
**4**	50	30	1	379.46	1772.54	4298.96	2831.25	52.06
**5**	50	30	2	382.49	1799.72	4578.43	3048.14	73.09
**6**	50	30	3	342.18	1489.73	4004.60	2857.70	66.37
**7**	0	30	1	347.03	1852.37	4279.49	2776.19	87.89
**8**	0	30	2	316.52	1769.14	3870.59	2503.28	65.24
**9**	0	30	3	324.37	1651.94	3915.26	2460.24	73.98
**10**	100	50	1	137.83	673.95	1565.33	1154.86	4.71
**11**	100	50	2	149.88	682.87	1726.26	1185.76	13.84
**12**	100	50	3	96.62	424.77	1126.23	679.50	6.35
**13**	50	50	1	348.95	1722.43	4179.84	3103.92	32.10
**14**	50	50	2	315.77	1511.81	3745.74	2462.17	38.69
**15**	50	50	3	342.00	1680.82	4135.17	2724.74	48.41
**16**	0	50	1	387.35	1862.56	4295.52	2701.89	63.79
**17**	0	50	2	348.63	1805.66	4397.46	2648.99	70.55
**18**	0	50	3	343.46	1696.05	4240.54	2755.75	76.28
**19**	100	70	1	151.39	776.29	2082.47	1269.19	25.09
**20**	100	70	2	146.13	723.63	2012.03	1177.71	27.88
**21**	100	70	3	126.15	608.13	1588.24	991.48	15.99
**22**	50	70	1	339.14	1764.90	4289.80	2816.10	42.45
**23**	50	70	2	363.94	1878.70	4666.63	2999.32	48.61
**24**	50	70	3	343.07	1734.32	4358.52	2662.94	55.20
**25**	0	70	1	371.83	1891.19	4522.31	2711.75	50.46
**26**	0	70	2	314.27	1574.66	4154.64	2475.63	58.03
**27**	0	70	3	357.91	1736.62	4440.99	2724.01	69.60
**28**	50	50	2	348.50	1785.40	4310.25	3012.45	68.40
**29**	50	50	2	332.15	1722.15	4185.60	2895.60	56.15
**30**	50	50	2	341.80	1758.60	4240.30	2954.20	62.30

Table S2 ANOVA analysis of the model.

Source	Sum of Squares	df	Mean Square	F-value	*p*-value	
			**Response 1: TPC [1]**			
**Source**	**Sum of Squares**	**df**	**Mean Square**	**F-value**	***p*-value**	
**Model**	276100.00	10	27609.25	66.87	< 0.0001	significant
A-EtOH/H_2O	190800.00	1	190800.00	462.06	< 0.0001*	
B-TEMPERATURE	45.09	1	45.09	0.1092	0.7447	
C-TIME	3262.17	1	3262.17	7.9	0.0112*	
AB	569.80	1	569.80	1.38	0.2546	
AC	140.63	1	140.63	0.3406	0.5663	
BC	530.80	1	530.80	1.29	0.271	
A^2	79099.79	1	79099.79	191.58	< 0.0001*	
B^2	337.48	1	337.48	0.8174	0.3773	
C^2	122.33	1	122.33	0.2963	0.5925	
ABC	55.76	1	55.76	0.135	0.7173	
**Residual**	7844.70	19	412.88			
Lack of Fit	7239.09	16	452.44	2.24	0.2766	not significant
Pure Error	605.61	3	201.87			
**Cor Total**	283900.00	29				
			**Response 2: ABTS [2]**			
**Source**	**Sum of Squares**	**df**	**Mean Square**	**F-value**	***p*-value**	
**Model**	7573000.00	10	757300.00	84.15	< 0.0001	significant
A-EtOH/H_2O	5501000.00	1	5501000.00	611.26	< 0.0001*	
B-TEMPERATURE	6928.61	1	6928.61	0.7699	0.3912	
C-TIME	147000.00	1	147000.00	16.34	0.0007*	
AB	2675.46	1	2675.46	0.2973	0.5919	
AC	4357.12	1	4357.12	0.4842	0.495	
BC	17845.57	1	17845.57	1.98	0.1752	
A^2	1810000.00	1	1810000.00	201.16	< 0.0001*	
B^2	8498.19	1	8498.19	0.9443	0.3434	
C^2	2664.17	1	2664.17	0.296	0.5927	
ABC	1764.48	1	1764.48	0.1961	0.6629	
**Residual**	171000.00	19	8999.23			
Lack of Fit	124500.00	16	7779.60	0.5018	0.8443	not significant
Pure Error	46511.78	3	15503.93			
**Cor Total**	7744000.00	29				
			**Response 3: DPPH [3]**			
**Source**	**Sum of Squares**	**df**	**Mean Square**	**F-value**	***p*-value**	
**Model**	42090000.00	10	4209000.00	87.2	< 0.0001	significant
A-EtOH/H_2O	29210000.00	1	29210000.00	605.21	< 0.0001*	
B-TEMPERATURE	240700.00	1	240700.00	4.99	0.0378*	
C-TIME	300000.00	1	300000.00	6.22	0.0221*	
AB	17372.87	1	17372.87	0.36	0.5556	
AC	92277.94	1	92277.94	1.91	0.1828	
BC	49570.17	1	49570.17	1.03	0.3236	

A^2	11630000.00	1	11630000.00	241.06	< 0.0001*	
B^2	202200.00	1	202200.00	4.19	0.0548	
C^2	49897.58	1	49897.58	1.03	0.322	
ABC	3106.30	1	3106.30	0.0644	0.8025	
**Residual**	917000.00	19	48262.51			
Lack of Fit	721900.00	16	45121.72	0.694	0.732	not significant
Pure Error	195000.00	3	65013.39			
**Cor Total**	43000000.00	29				
			**Response 4: FRAP [4]**			
**Source**	**Sum of Squares**	**df**	**Mean Square**	**F-value**	***p*-value**	
**Model**	17940000.00	10	1794000.00	64.77	< 0.0001	significant
A-EtOH/H_2O	10580000.00	1	10580000.00	382.04	< 0.0001*	
B-TEMPERATURE	1199.03	1	1199.03	0.0433	0.8374	
C-TIME	200100.00	1	200100.00	7.23	0.0146*	
AB	4467.56	1	4467.56	0.1613	0.6924	
AC	66382.66	1	66382.66	2.4	0.1381	
BC	5640.70	1	5640.70	0.2037	0.6569	
A^2	6751000.00	1	6751000.00	243.72	< 0.0001*	
B^2	3498.55	1	3498.55	0.1263	0.7262	
C^2	1652.17	1	1652.17	0.0596	0.8097	
ABC	5866.61	1	5866.61	0.2118	0.6506	
**Residual**	526300.00	19	27697.75			
Lack of Fit	337900.00	16	21121.64	0.3365	0.9369	not significant
Pure Error	188300.00	3	62770.34			
**Cor Total**	18470000.00	29				
			**Response 5: BCB [5]**			
**Source**	**Sum of Squares**	**df**	**Mean Square**	**F-value**	***p*-value**	
**Model**	17414.43	10	1741.44	20.01	< 0.0001	significant
A-EtOH/H_2O	14385.47	1	14385.47	165.25	< 0.0001*	
B-TEMPERATURE	82.01	1	82.01	0.942	0.3439	
C-TIME	198.40	1	198.40	2.28	0.1476	
AB	916.65	1	916.65	10.53	0.0043*	
AC	30.24	1	30.24	0.3474	0.5625	
BC	22.03	1	22.03	0.2531	0.6207	
A^2	1236.37	1	1236.37	14.2	0.0013*	
B^2	89.64	1	89.64	1.03	0.323	
C^2	111.79	1	111.79	1.28	0.2712	
ABC	291.37	1	291.37	3.35	0.0831	
**Residual**	1653.95	19	87.05			
Lack of Fit	1161.44	16	72.59	0.4422	0.8795	not significant
Pure Error	492.52	3	164.17			
**Cor Total**	19068.38	29				

[1] Total Phenolic Content (TPC); [2] 2,2′-azino-bis(3-ethylbenzothiazoline-6-sulfonic acid) (ABTS); [3] 2,2-diphenyl-1-picrylhydrazyl (DPPH); [4] Ferric Reducing Antioxidant Power (FRAP); [5] *β*-Carotene Bleaching assay (BCB); df: Degree of freedom; *p*-value < 0.05 was considered significant.

Table S3. Fit statistics of the model.

	TPC [1]	ABTS [2]	DPPH [3]	FRAP [4]	BCB [5]
**Std. Dev.**	20.32	94.86	219.69	166.43	9.33
**Mean**	284.97	1411.69	3476.60	2269.43	45.55
**C.V. %** [6]	7.13	6.72	6.32	7.33	20.48
**R^2**	0.97	0.98	0.98	0.97	0.91
**Adjusted R^2**	0.96	0.97	0.97	0.96	0.87
**Predicted R^2**	0.94	0.95	0.95	0.94	0.81
**Adeq Precision**	20.80	24.26	23.87	20.91	14.95

[1] Total Phenolic Content (TPC); [2] 2,2′-azino-bis(3-ethylbenzothiazoline-6-sulfonic acid) (ABTS); [3] 2,2-diphenyl-1-picrylhydrazyl (DPPH); [4] Ferric Reducing Antioxidant Power (FRAP); [5] β-Carotene Bleaching assay (BCB); C.V.%: coefficient of variation.

 https://doi.org/10.3390/xxxxx

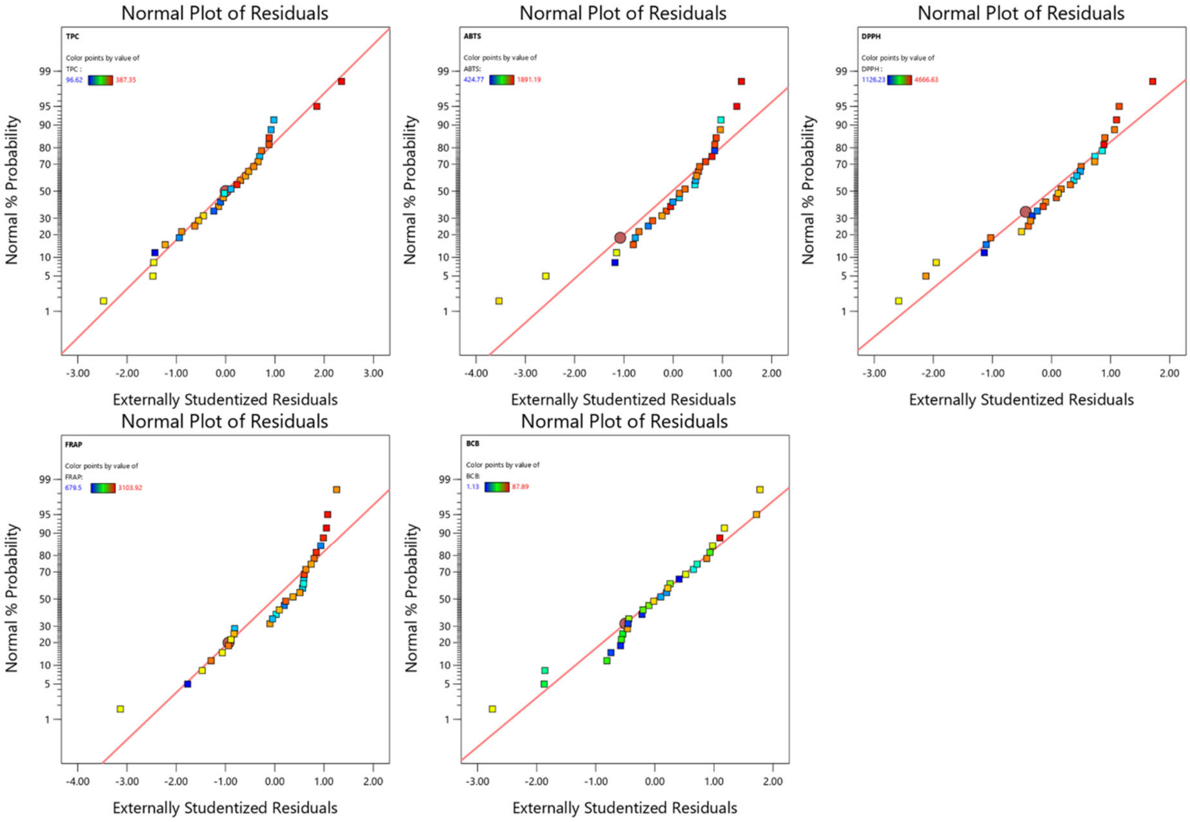

Figure S1. Normal Plot of residuals for Total Phenolic Content (TPC), 2,2′-azino-bis(3-ethylbenzothiazoline-6-sulfonic acid) (ABTS), 2,2-diphenyl-1-picrylhydrazyl (DPPH), Ferric Reducing Antioxidant Power (FRAP), and *β*-Carotene Bleaching assay (BCB).
